# Nonnutritive sweeteners and glucose intolerance: Where do we go from here?

**DOI:** 10.1172/JCI171057

**Published:** 2023-05-15

**Authors:** Samuel Philip Nobs, Eran Elinav

**Affiliations:** 1 Systems Immunology Department, Weizmann Institute of Science, Rehovot, Israel.; 2 Division of Microbiome and Cancer, Deutsches Krebsforschungszentrum (DKFZ), Heidelberg, Germany.

Cardiometabolic disease, including obesity, type 2 diabetes, hyperlipidemia, and nonalcoholic fatty liver disease, is clearly associated with high sugar intake. In an attempt to provide a low-calorie alternative to food sweetening, nonnutritive sweeteners (NNSs) are increasingly consumed by millions of individuals, but their long-term impact on human health in general and on metabolism in particular is not yet fully understood. In contrast to earlier assumptions that these hypersweet compounds may be inert to the human body, accumulating evidence suggests that NNS consumption may feature a profound but individualized impact on human metabolism through their capacity to modulate both host and microbiome (1). However, a mechanistic understanding of NNS effects on human physiology is lacking or incomplete in most cases, and a concerted medical and scientific effort is needed to clarify the potential long-term implications of NNS consumption on human health (Figure 1). Given the multitude of reports on the lack of NNS inertness, the burden of proof has shifted from a need to prove that NNSs are unsafe to a necessity of understanding their potential scope of effects on humans in order to optimize their recommended use by populations at risk. In this Viewpoint, we focus on the documented influences of NNSs on glycemic responses in exemplifying some of the key considerations and open questions challenging the exploration of the NNS effects on metabolic health, while suggesting approaches that may meet these challenges. Other suggested associations between NNS usage and nonmetabolic human disease, such as recently reported effects on acute cardiovascular events (2), merit similar considerations but are beyond the scope of this Viewpoint.

Human consumption of NNSs as an alternative to caloric sugars has been followed and debated for decades. Examples of the rich, yet hotly contested literature on the matter include some reports indicating no effect of NNS consumption on metabolic parameters (3) and others suggesting both positive (4) and negative effects of NNS usage on metabolic health (5). Interpretation of these studies is often challenging due to differences in methodology, personalized variations in responses to NNS, and the fact that many of the studies are observational rather than prospective, randomized clinical trials (RCTs). Studies in animal models have aimed to clarify these controversies, while demonstrating causality and mechanism, but have often been similarly confusing. While many animal studies suggest negative effects of NNS intake on metabolic features (6), some reported no impact on metabolic parameters (7), while others demonstrated positive effects on readouts such as body weight, fat mass, and waist circumference (4). Importantly, animal experiments have allowed unraveling some of the potential underlying mechanisms driving NNS effects on human physiology. Examples include indications of potential causal effects of some NNSs on the gut microbiome driving an altered host metabolism (6) and suppressive NNS effects on adaptive immunity in the context of cancer and infection (8). Careful consideration of the factors contributing to the above ambiguities in animal and human results may enable optimization of research toward better crystallization of NNS effects on human metabolism and associated mechanisms of activity.

## Variations in NNS chemical structure and downstream mechanisms

One of the key complexities in studying the impact of NNS on metabolic health relates to the fact that these intensely sweet compounds comprise a group of distinct molecules with potentially different chemistries that thus may affect glucose intolerance and other metabolic parameters in unique manners. For example, sucralose is a disaccharide, and saccharin is a benzisothiazole, while aspartame is a methyl ester. The difference in molecular structure influences NNS metabolism, modifications, and host reactivity. NNSs such as aspartame are broken down into their components aspartic acid, phenylalanine, and methanol and thus could potentially exert multiple effects on the host through these metabolic breakdown products (9). Other NNSs, such as saccharin or sucralose, are believed to pass through the gastrointestinal tract with little modification, exhibiting only a small proportion of metabolic breakdown products (10). Acesulfame potassium, on the other hand, is not metabolized and is rapidly excreted (11). Taken together, these data show that NNSs may mediate their biological effects both directly and through effects exerted by their degradation products.

To exemplify this mechanistic complexity, even NNS interaction with oropharyngeal sweet taste receptors is mediated through binding to different domains on these receptors (9). Importantly, NNSs also bind sweet-taste receptors in the gastrointestinal tract, with potentially different effects exerted by those interactions on host metabolism. Interestingly, these gastrointestinal sweet taste receptors are mainly expressed in enteroendocrine cells that are a major source of hormones. However, no consistent results in experimental models or in humans prove to date a direct capacity of NNSs to induce in vivo production of hormones such as GLP-1 (9). To further illustrate the complexity, sucralose (12) and stevia (13) consumption were associated with increased levels of insulin, similar to that noted during consumption of glucose, but the mechanisms driving this effect remain unclear. Interestingly, mouse studies suggest that stevia may directly promote insulin secretion by β cells (14) via the stimulation of the TRPM5 receptor (15). Other studies have also shown that some NNSs, such as sucralose, acesulfame-K, and saccharin, may upregulate intestinal glucose uptake (16). Investigating how responses to carbohydrates and NNSs uniquely intersect will be critical to better understanding the disparate metabolic effects of NNSs on human health.

## Host dietary, microbial, and metabolic states

A critical element in understanding the impact of NNS intake on metabolic health, while explaining some of the phenotypic differences noted between studies, relates to the baseline metabolic state of individuals consuming NNSs. Some human NNS studies have focused on healthy individuals (13), while others evaluated NNSs in diabetic or obese individuals (17), which may affect key metabolic parameters, such as glucose intolerance, body weight, intestinal barrier integrity, and low-grade inflammation (18). Additionally, a leakier gut epithelial barrier in people with cardiometabolic disease may lead to increased systemic spread of these molecules, which in turn could enhance their global metabolic impact. Furthermore, due to the profound effect of diet on the composition of the microbiome (as highlighted below), differences in microbial composition associated with cardiometabolic disease are likely to affect degradation of NNSs in the intestinal tract, which in turn will lead to different levels of NNSs and NNS-derived metabolites in different clinical settings.

## Short-term versus long-term NNS consumption

A critical aspect, possibly complicating interpretation of NNS studies, relates to the duration of human NNS exposure. While several RCTs (13, 17) have demonstrated a significant impact of short-term consumption of some NNSs, such as sucralose or saccharin, on glycemic responses, whether continuous NNS intake for longer periods of time may further enhance or alternatively mitigate these effects remains elusive to date. Animal models suggest that long-term consumption of some NNSs may be associated with additional adverse health features, such as impaired cognition (19), liver damage (20), and disrupted circadian rhythms (21), but it remains unknown to what extent these findings can be translated to humans.

## NNS interactions

NNS modulatory effects on metabolism may depend on concomitant coconsumption of other dietary compounds. For example, habitual consumption of sucralose may affect glucose metabolism in a stronger manner if it is taken with carbohydrates (22). The differences between NNS consumption in their purified forms (such as in dietary beverages) versus consumption as combinations with glucose in sachets merit future studies. Relatedly, the effects of NNSs on individuals following a low-carbohydrate diet may differ from that on those consuming high-carbohydrate diets, meriting future exploration. Additionally, humans may habitually consume multiple different types of NNSs in the form of dietary beverages, contents of sachets, and NNSs embedded within multiple food products. The food industry uses at times sweetener blends in sweetened food products, in order to mask the bitter aftertaste of individual sweeteners (23). Such putative interactions and related effects of NNS modulation of the human host merit future studies.

## Correlation versus causation

Determining causal associations between NNS consumption and metabolic outcomes constitutes a major challenge. Many observational studies have noted associations between NNS intake and metabolic derangements, without being able to identify what is the cause and what the consequence of such correlations (1). This issue of reverse causality, namely whether individuals with NNS intake are more likely to develop cardiometabolic disease, or alternatively whether people who suffer from features of cardiometabolic disease opt to consume more NNSs (with a hope of improving their disease manifestations) can be resolved by prospective and well-controlled RCTs. Of note, such studies should be carefully planned and executed, while avoiding pitfalls such as underpowering due to interindividual variability and the highly prevalent and often unaware exposure of consumers to NNS-embedded foods, which may create major biases in study results. For example, in one recent RCT, a careful nutritional assessment led to the exclusion of 1,244 out of 1,375 eligible participants solely based on unaware regular nutritional exposure to NNS (13).

## Microbiome-NNS interactions

Possible effects of NNS on the composition and function of the microbiome have emerged as important elements, possibly affecting NNS modulation of host metabolism. Additionally, interindividual variability in microbiome composition and function may help to resolve some of the ambiguities previously noted among NNS studies. While both rodents (6) and humans (13) demonstrate reproducible NNS effects on microbiome configurations, the causative commensals, their mechanisms of NNS sensing and response, and conditions driving these microbiome changes remain elusive and merit future studies. NNSs may influence microbes through multiple distinct mechanisms, including modulating interbacterial communication via quorum sensing (24), affecting genome integrity (25), altering nutrient import/export (26), or affecting the structure of the bacterial membrane (27), among others. Furthermore, NNSs may directly regulate growth of bacteria (26), including promoting expansion of specific bacterial species in vivo (28). Importantly, causality of NNS-modulated microbiome effects on host glycemic responses was suggested via a large series of fecal transfer experiments from humans consuming saccharin, sucralose, aspartame, and stevia (or their respective controls) into germ-free mice (13). These demonstrated that mouse recipients of human microbiomes from NNS consumers largely mirrored the glycemic responses of their human donors. As such, microbiome transfers from humans featuring NNS-induced glycemic alterations caused similar alterations in recipient mice, while microbiome transfers from humans not reacting to NNSs resulted in little or no glycemic alterations in recipient mice. Importantly, NNS-induced alterations on the human microbiome featured unique signatures, compatible with their differences in chemical structures. A gradient in the metabolic effects of NNSs on the microbiome and downstream host metabolism was noted. Saccharin and sucralose induced the most significant metabolic and microbiome perturbations, while aspartame and stevia affected some, but not all individuals. The mechanisms driving these changes and their persistence, reversibility, and unique effects along the human gastrointestinal tract (29), merit future studies. Likewise, possible NNS effects on other microbiome kingdoms, such as fungi and eukaryotes, may constitute interesting prospects for future research.

## Concluding remarks

The medical and scientific communities are only beginning to achieve a comprehensive mechanistic understanding of the possible effects of NNS consumption on glucose intolerance and metabolic health. Disentangling the effects of duration and dosage of NNS exposure, as well as a multitude of other varying host, dietary, and microbial factors that may possibly modulate host reactivity to NNS may enable us to move beyond correlative descriptions into demonstration of causality and mechanism. Such causal understanding is essential in enabling individualized optimization of NNS use, development of newer generations of sweeteners, and assurance of their safety.

## Figures and Tables

**Figure 1:**
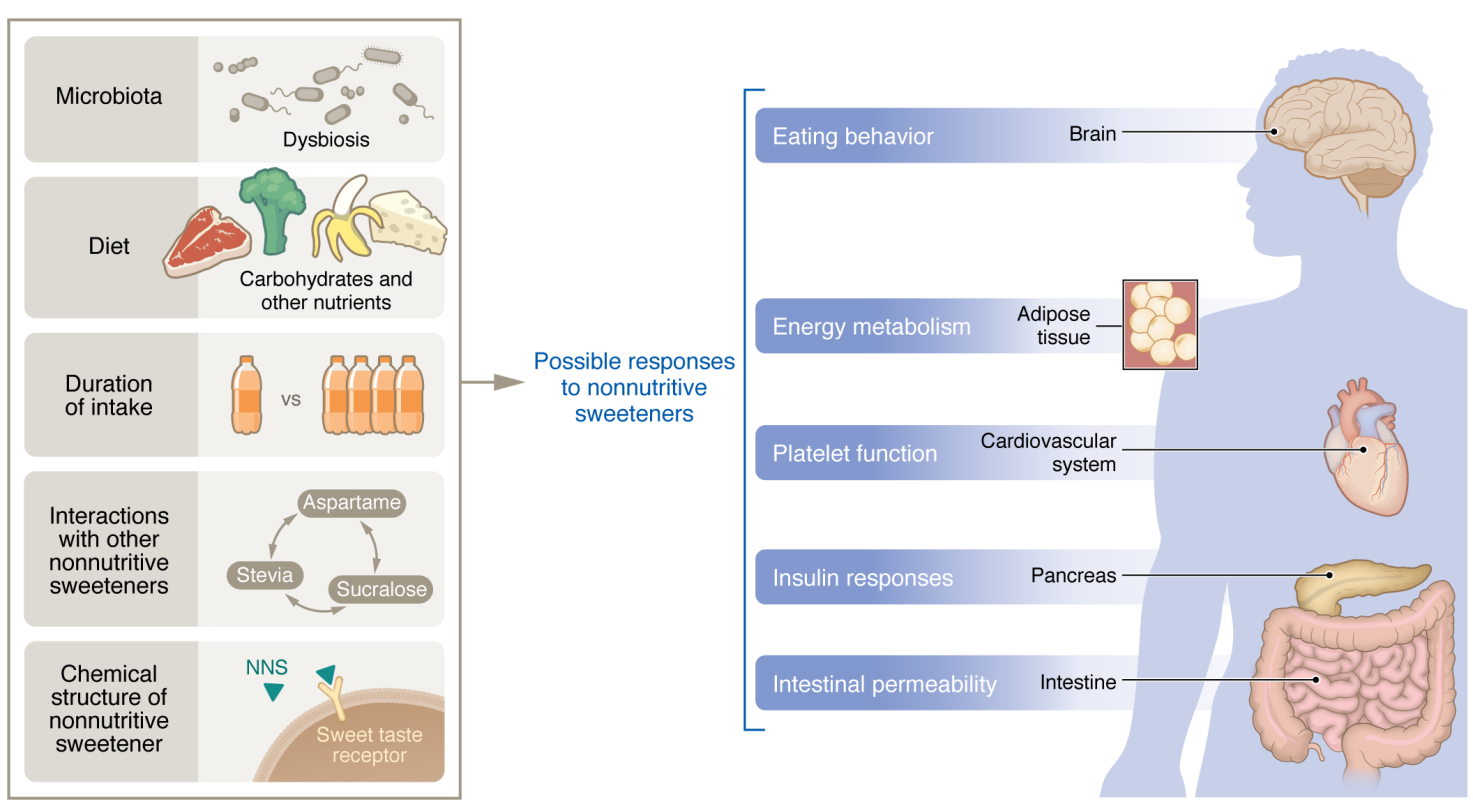
The possible responses to NNSs. An overview of how different factors may affect the physiological impact of NNS consumption.

## Version 1 05/15/2023

Electronic publication
